# Breast Milk Microbiota Is Shaped by Mode of Delivery and Intrapartum Antibiotic Exposure

**DOI:** 10.3389/fnut.2019.00004

**Published:** 2019-02-04

**Authors:** Henriina Hermansson, Himanshu Kumar, Maria Carmen Collado, Seppo Salminen, Erika Isolauri, Samuli Rautava

**Affiliations:** ^1^Department of Paediatrics, University of Turku and Turku University Hospital, Turku, Finland; ^2^Functional Foods Forum, University of Turku, Turku, Finland; ^3^Institute of Agrochemistry and Food Technology (IATA-CSIC), Spanish National Research Council, Valencia, Spain

**Keywords:** breast milk, microbiota composition, cesarean section, intrapartum antibiotics, non-communicable diseases

## Abstract

The mode of delivery has been suggested to modulate the bacterial composition of breast milk but the impact of intrapartum antibiotic use on the milk microbiota is currently not known. The aim of this study was to analyze the effects of the mode of the delivery and intrapartum antibiotic administration on the microbial composition of breast milk. Breast milk samples were collected from 84 healthy mothers 1 month after the delivery. In total, 61 mothers had delivered vaginally, 23 of which had received intrapartum antibiotics, 13 women had delivered with non-elective cesarean section, 7 of which had received antibiotics, and 10 mothers had delivered with elective cesarean section without intrapartum antibiotic treatment. Both mode of delivery and intrapartum antibiotic exposure were significantly associated with changes in the milk microbial composition as assessed by analysis of similarities (ANOSIM) test (*p* = 0.001). The mode of delivery had a more profound effect on the milk microbiota composition as compared to intrapartum antibiotic exposure. Although the clinical significance of breast milk microbiota is currently poorly understood, this study shows that cesarean section delivery has an independent effect on breast milk microbiota composition. The dysbiosis observed in infants born by cesarean section delivery may be aggravated by the aberrant breast milk microbiota.

## Introduction

Human breast milk is considered the gold standard of infant nutrition. Beyond its nutritional benefits, breastfeeding is known to reduce respiratory and gastrointestinal infections in early life, and decrease the risk of non-communicable diseases including type 2 diabetes mellitus, overweight and obesity in childhood as well as improve neuro-developmental outcomes particularly in preterm infants (1). The protective mechanisms of breastfeeding may partly be explained by its impact on the composition of gut microbiota. Breast milk contains several components, including oligosaccharides and glycoproteins, which modulate the neonatal gut microbiota composition by favoring the growth of specific bifidobacteria (2). In addition, breast milk harbors a unique microbiota which might also serve as a continuous source of colonizing bacteria to the newborn infant (3–5).

Infants born by cesarean section display differences in gut microbiota composition and immune responses as compared to infants born by vaginal delivery [reviewed by (6)]. In a similar fashion, intrapartum antibiotic exposure has been reported to perturb infant gut colonization [reviewed by (7)]. Recent studies suggest that the mode of delivery shapes the bacterial composition of breast milk (8, 9) but the impact of intrapartum antibiotic use on the milk microbiota is currently not known. Antibiotic prophylaxis is commonly used during both elective and non-elective cesarean section deliveries but the contribution of antibiotic exposure to the milk microbiota perturbations associated with cesarean section delivery has not previously been investigated. The objective of this study was to analyze the effects of the mode of delivery and intrapartum antibiotic administration during delivery on the microbial composition of breast milk.

## Subjects and Methods

The mothers in this study were selected based on breast milk sample availability from a randomized clinical trial (10) assessing the impact of maternal probiotic supplementation on the occurrence of atopic dermatitis. In the original study, a probiotic combination consisting of either *Lactobacillus rhamnosus* LPR and *Bifidobacterium longum* BL999 or *Lactobacillus paracasei* ST11 and *Bifidobacterium longum* BL999 or placebo was administered beginning 2 months before the expected delivery and continued until 2 months after delivery. Altogether, 38 women who had delivered vaginally without intrapartum antibiotic exposure, 23 women who had delivered vaginally and received intrapartum antibiotics, 10 women who had delivered by elective cesarean section without intrapartum antibiotic exposure, 6 women who had delivered with non-elective cesarean section without intrapartum antibiotics and 7 women who had delivered with non-elective cesarean section and received intrapartum antibiotics were selected for this study. All the women in the study delivered after 36 weeks of gestation after an uncomplicated pregnancy. Written informed consent was obtained from all participants. The study was deemed ethically acceptable by the Ethics Committee of the Hospital District of Southwest Finland.

Maternal, pregnancy and birth data were collected from hospital records. The breast milk samples were collected at home 1 month after delivery. Mothers received written instructions for standardized self collection of samples in the morning. Before the sample collection, the breast was cleaned and breast milk was collected manually, discarding the first drops, with a sterile milk collection unit. The milk samples were collected between August 2005 and April 2009 and stored at −20°C for later analyses.

Breast milk samples were processed for DNA isolation in the spring of 2016 using our previously established methods as described in detail elsewhere (4). Purified DNA was used for 16S rRNA gene amplification, multiplexing was carried out using Nextera XT Index kit (Illumina, CA, USA). Primers targeting the V3-V4 region of 16S rRNA gene were used for amplification according to previously described methods using 2 × 300 bp paired-end run Illumina MiSeq platform (FISABIO sequencing service, Valencia, Spain) (11).

Quality assessment was performed using the prinseq-lite program (min_length:50; trim_qual_right:20; trim_qual_type:mean; trim_qual_window:20) (12). Filtered and demultiplexed sequences were processed using open source software QIIME (version 1.9.1, with default parameters) (13). The sequences were clustered to form OTUs tables (97% identity), and taxonomy classification was obtained at phylum, family, genus levels by use of Greengenes 13_8database. Alpha diversity indices (Shannon index and richness index) were calculated. Calypso version 8.4 (http://cgenome.net/calypso/) was used with total sum normalization (TSS) and square root transformed, for the multivariate analysis using OTU phylotype. *P* ≤ 0.05 were regarded as statistically significant.

## Results

The clinical characteristics of study subjects are presented in Table 1. Briefly, there were no differences in maternal pre-pregnancy weight, body mass index, or weight gain during pregnancy between the delivery groups. Gestational diabetes mellitus was observed in 14 (17%) mothers. Altogether 19 (23%) mothers reported smoking before pregnancy and 3 (4%) continued smoking during pregnancy. The intervention received by the mothers in the probiotic intervention trial (10) is presented in Table 1.

**Table 1:** The clinical characteristics of the study population.

	**VD without IAP *n* = 38**	**VD with IAP *n* = 23**	**Non-elective CS without IAP *n* = 6**	**Non-elective CS with IAP *n* = 7**	**Elective CS without IAP *n* = 10**	**Total *n* = 84**
Mother's age (years)	32 (26**–**44)	31 (23**–**40)	31 (29**–**34)	32 (30**–**34)	32 (28**–**39)	32 (23**–**40)
First pregnancy (yes)	15 (60%)	21 (58%)	5 (83%)	6 (85%)	6 (60%)	53 (63%)
Pre-pregnancy BMI (kg/m^2^)	23.4 (18.4**–**33.6)	24.3 (19.7**–**38.5)	23.0 (20.0**–**27.0)	24.9 (22.4**–**28.2)	25.6 (20.2**–**33.5)	24.1 (18.4**–**38.5)
Maternal weight gain (kg)	15.7 (9.1**–**25)	14.4 (5.3**–**28.5)	13.7 (3.1**–**23.3)	17.2 (11.5**–**26.3)	13.3 (6,4**–**18.6)	14.4 (3.1**–**28)
Gestational diabetes (yes)	3 (8%)	9 (40%)	1 (17%)	0 (0%)	1 (10%)	14 (17%)
Maternal IAP						
-Penicillin G		18		–		18
-Cephalotin		2		3		5
-Cephalexin		1		–		1
-Penicillin G and metronidazole		–		1		1
-Cefuroxim and metronidazole		–		3		3
-Cefuroxim		1				1
Infant gender male/female	17/19	17/8	5/1	3/4	9/1	51/33
Gestational age (weeks)	40.7 (35.7–42.3)	39.8 (37.6–41.9)	40.0 (36.6–41.1)	40.8 (38.1–42.4)	39.1 (38.1–39.3)	39.8 (35.7–42.4)
Birth weight (g)	3540 (4460–2430)	3420 (2850–4100)	3770 (2860–4660)	3920 (3190–4650)	3560 (2910–4030)	3550 (4660–2430)
Birth length (cm)	50.3 (46–56)	50.6 (48–54)	53 (52.6)	52 (50–53)	50 (45–54)	51 (45–56)
Head circumference (cm)^*^	35 (32–39)	35 (33–37)	36 (35–37)	35 (33–37)	37 (35–37)	35 (32–39)
Weight at 6 months (kg)	8.2 (6.4–12.4)	8.3 (6.3–10.2)	8.2 (7.0–9.3)	8.0 (6.2–9.4)	8.2 (7.0–9.7)	8.2 (6.2–12.4)
Length at 6 months (cm)	67.8 (63.0–75.5)	68.4 (64.0–72.2)	69.3 (67.3–72.8)	67.8 (63.1–71.6)	67.4 (63.6–72.3)	68 (63.0–76.0)
Head circumference at 6 months (cm)	43.8 (40.7–48.3)	43.9 (42.0–46.2)	44.7 (43.3–45.5)	43.6 (41.0–45.3)	44.0 (40.7–48.3)	44.0 (40.7–48.3)
Neonatal antibiotic treatment	1 (3%)	0 (0%)	1 (17%)	0 (0%)	1 (10%)	3 (4%)
Exclusive breastfeeding at 1mo (yes)	29 (80%)	17 (68%)	4 (57%)	3 (50%)	6 (60%)	59 (70%)
Probiotics						
-LPR+BL999^**^	10	9	2	10	2	33
-ST11+BL999^***^	9	5	2	9	3	28
-Placebo	17	11	2	17	5	52

*Continuous variables are expressed as a mean and range. (VD, vaginal delivery; IAP, intrapartum antibiotic prophylaxis; CS, cesarean section delivery)*.

* *Statistically significant difference between the delivery groups (P = 0.007)*.

** *Lactobacillus rhamnosus LPR and Bifidobacterium longum BL999*.

*** *Lactobacillus paracasei ST11 and Bifidobacterium longum BL999*.

In the vaginal delivery group, the intrapartum antibiotics used included penicillin G (18 mothers), cephalothin (2 mothers), cephalexin (1 mother), or cefuroxime (1 mother). In the non-elective CS group 3 mothers received cephalothin, 3 mothers received cefuroxime combined with metronidazole and one mother received penicillin G combined with metronidazole. As per the obstetric guidelines of the time, none of the mothers undergoing elective cesarean section received antibiotic prophylaxis before the surgery.

The duration of pregnancy was shorter in the elective cesarean section group (mean 39.9 weeks, range 39–42) compared to the vaginal delivery and non-elective cesarean section groups (mean 40.2 weeks, range 36–42 and 41.4 weeks, range 37–43), respectively, *P* = 0.042.

**Figure 1:**
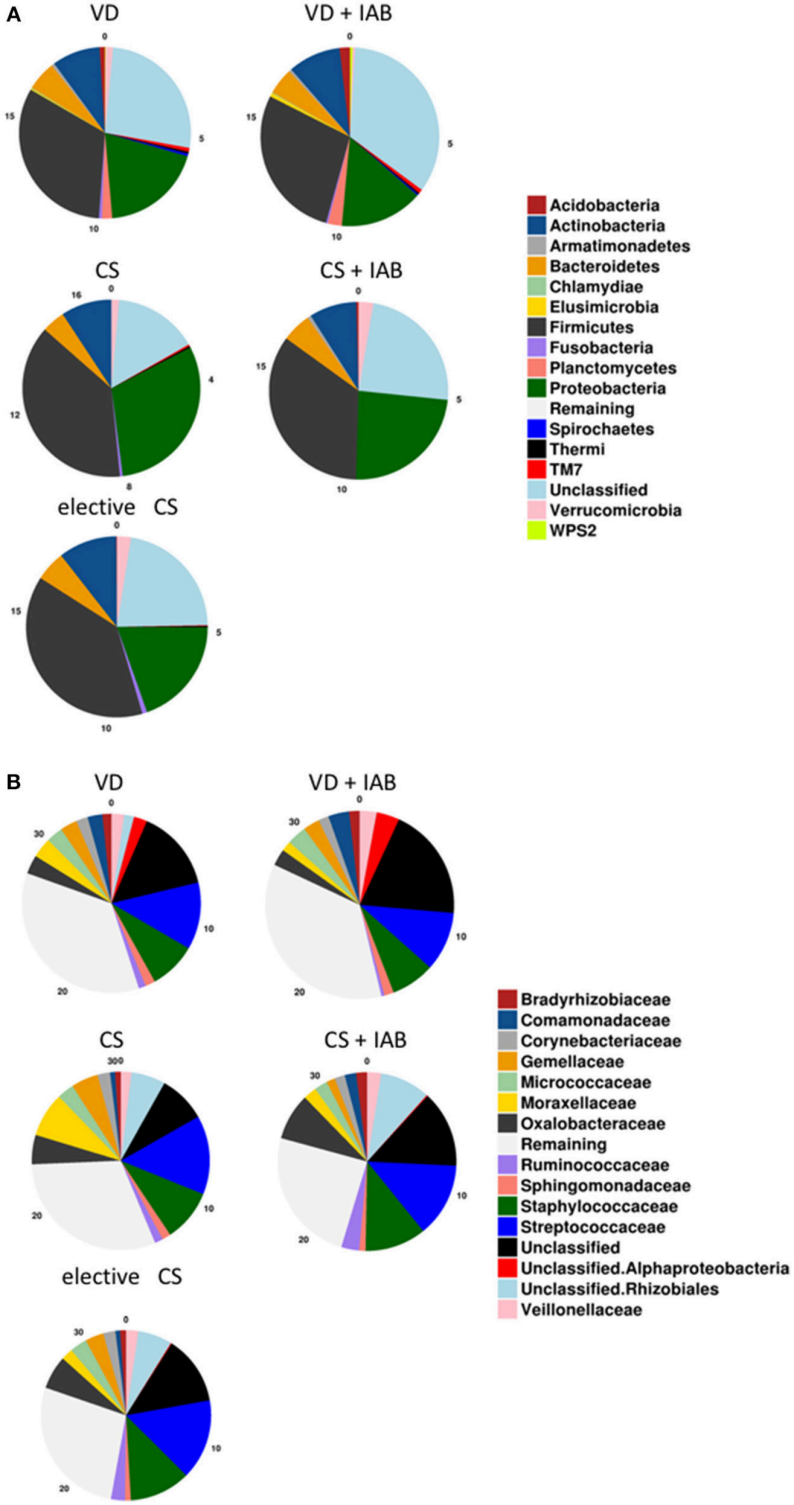
Breast milk microbiota composition 1 month after delivery in mothers who delivered vaginally (VD) or by cesarean section (CS) and exposed or not exposed to intrapartum antibiotics (IAB). The relative abundance of phyla **(A)** and the 15 most abundant families **(B)** are presented.

The most abundant phyla in the milk microbiota 1 month after delivery were Proteobacteria and Firmicutes (Figure 1A). No statistically significant differences related to the mode of delivery or intrapartum antibiotic exposure were detected in the relative abundance of bacteria on the phylum level. *Streptococcaceae* and *Staphylococcaceae* were the most abundant bacterial families (Figure 1B). No consistent statistically significant differences related to mode of delivery or intrapartum antibiotic exposure were detected in the most abundant bacterial families.

**Figure 2:**
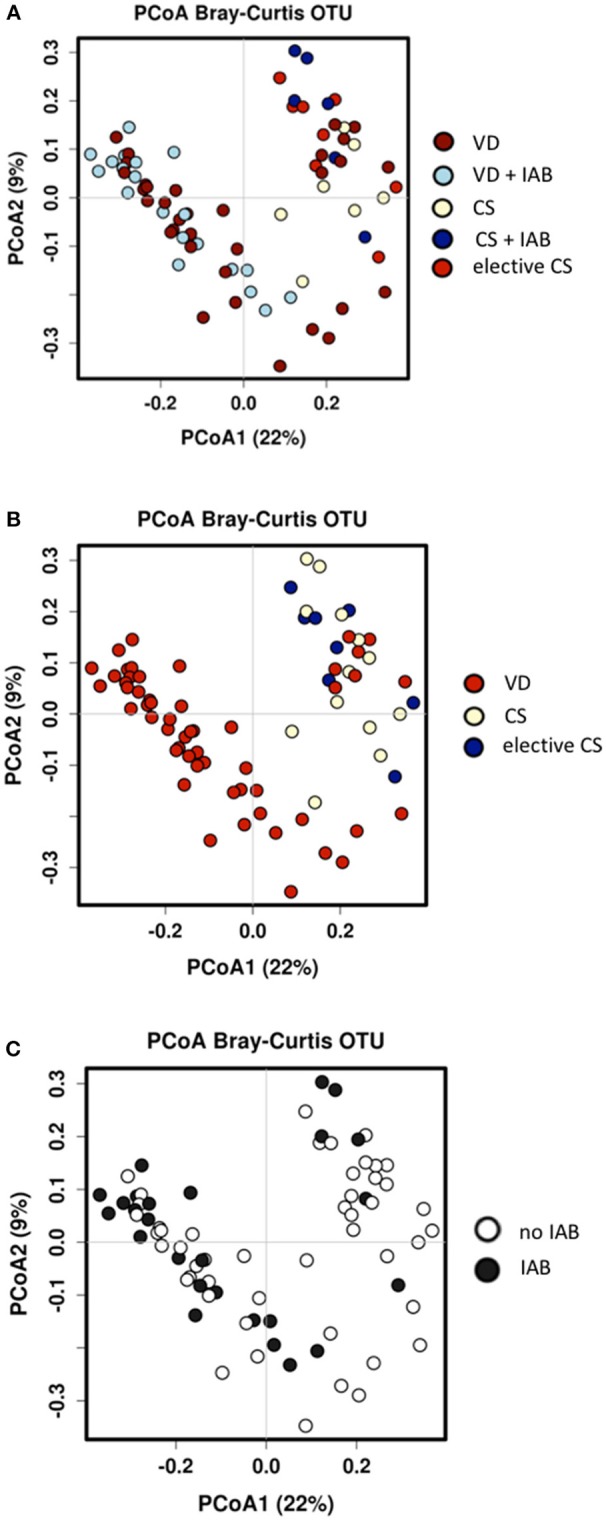
Significant differences in breast milk microbiota composition were detected 1 month after delivery between mothers who delivered vaginally (VD) or by cesarean section (CS) and exposed or not exposed to intrapartum antibiotics (IAB) by Bray-Curtis Principal coordinate analysis (PCoA) and ANOSIM test; *p* = 0.001 **(A)**. When the mothers were grouped by only mode of delivery, VD mothers clustered distinctly from CS mothers; ANOSIM *p* = 0.001 **(B)**. In contrast, no significant clustering was detected when the mothers were group by IAB; ANOSIM *p* = 0.54 **(C)**.

Both mode of delivery and intrapartum antibiotic exposure were associated with changes in the overall microbial composition in breast milk samples collected 1 month after delivery. The total number of sequences was around 700 k with mean sequence counts per subject at around 10,000 reads. Marked clustering of the study groups was detected by Principal Coordinate Analysis (PCoA) of the breast milk microbiota (Figure 2A) and the observation was statistically significant as assessed by analysis of similarities (ANOSIM) test (*p* = 0.001). When the mothers were grouped solely by mode of delivery, significant differences in microbiota profiles were observed according to the PCoA (Figure 2B). The breast milk microbiota of mothers who had delivered vaginally was significantly distinct from that of mothers who had delivered by cesarean section. In contrast, intrapartum antibiotic exposure as the solely variable was not associated with statistically significant changes in breast milk as assessed by ANOSIM (Figure 2C). Redundancy discrimination analysis (RDA) was used to independently analyze the impact of delivery mode and intrapartum antibiotics on the breast milk microbiota. When these explanatory factors were considered simultaneously in the analysis, both mode of delivery (*p* = 0.001) and intrapartum antibiotic exposure (*p* = 0.015) were found to be independently associated with the breast milk microbiota composition 1 month after delivery. No statistically significant differences in the overall breast milk microbiota composition as assessed by ANOSIM were detected with regard to the probiotic intervention (Supplementary Figure 1A). When probiotic intervention, mode of delivery and intrapartum antibiotic exposure were all simultaneously included as explanatory factors in RDA analysis, it was apparent that probiotic intervention had no effect on the breast milk microbiota (*p* = 0.73) and mode of delivery (*p* = 0.001) and intrapartum antibiotics (*p* = 0.009) are responsible for the observed breast milk microbiota differences.

**Figure 3:**
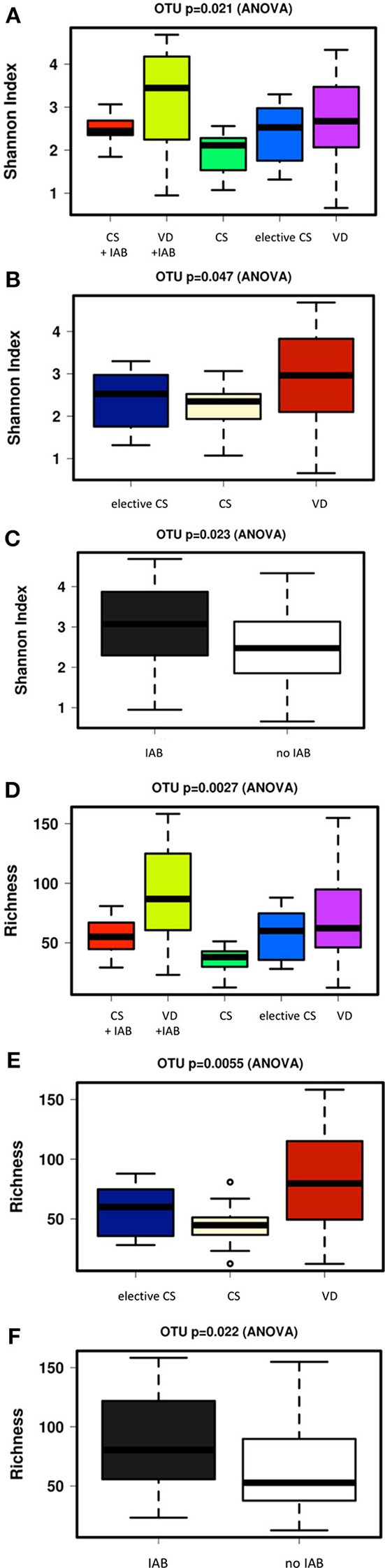
Significant differences in breast milk microbiota diversity as assessed by Shannon index were observed 1 month after delivery between mothers who delivered vaginally (VD) or by cesarean section (CS) and exposed or not exposed to intrapartum antibiotics (IAB) **(A)**. VD mothers exhibited significantly higher breast milk microbiota diversity as compared to CS mothers **(B)** while IAB was associated with higher microbiota diversity in breast milk **(C)**. In a similar fashion, breast milk microbiota richness varied significantly depending on birth mode and IAB **(D)**. Breast milk microbiota richness was significantly higher in VD mothers compared to CS mothers **(E)**. IAB was associated with increased breast milk microbiota richness **(F)**.

The alpha diversity of the breast milk microbiota was significantly higher in mothers who had delivered vaginally as compared with the mothers who had delivered by cesarean section (Figures 3A,B). Intrapartum antibiotic treatment, on the other hand, was associated with higher breast milk microbiota alpha diversity when analyzed as a single variable (Figure 3C). In a similar fashion, the microbiota richness was significantly higher in the breast milk of mothers who had delivered vaginally as compared to those who had delivered by cesarean section (Figures 3D,E), whereas intrapartum antibiotic exposure was associated with significantly higher breast milk microbiota richness (Figure 3F). The breast milk microbiota alpha diversity and richness were comparable between the mothers who received placebo or probiotics in the clinical trial (Supplementary Figures 1B,C).

**Figure 4:**
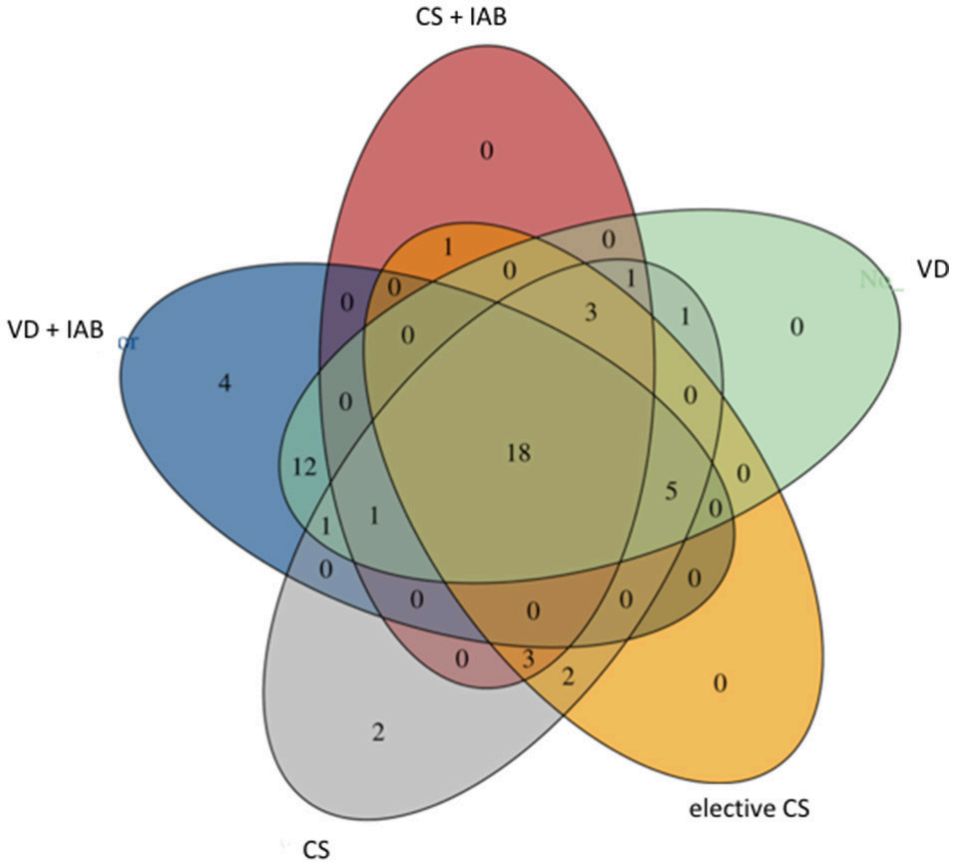
Venn diagram presenting shared bacterial families in breast milk microbiota in mothers who delivered vaginally (VD) or by cesarean section (CS) and exposed or not exposed to intrapartum antibiotics (IAB).

A core breast milk microbiota comprising of 18 bacterial families was shared between the mothers despite differences in the mode of delivery and intrapartum antibiotic exposure (Figure 4). In accordance with a previous report (4), *Streptococcaceae, Staphylococcaceae*, and *Bifidobacteriaceae* families were among the constituents of the core microbiome. It is of note that 16 bacterial families were detected only in the breast milk of mothers who delivered vaginally (Figure 4). Interestingly, when the milk samples were separated based on antibiotic exposure, the species *Bifidobacterium* was uniquely found in the breast milk samples of mothers who did not receive antibiotics.

## Discussion

Both mode of delivery and exposure to intrapartum antibiotics had an independent impact on the composition of the breast milk microbiota at 1 month *postpartum* in this study. A previous study by Cabrera-Rubio and colleagues has indicated that cesarean section delivery and particularly elective cesarean section is associated with altered breast milk microbial composition (8). This has been interpreted to suggest that labor might trigger increased intestinal permeability, enhanced bacterial translocation in the maternal gut and consequently transfer of bacteria to breast milk. Transport of intestinal bacteria or ingested probiotics to breast milk has previously been reported from clinical studies (14, 15). Intrapartum antibiotic prophylaxis, which is recommended and commonly administered prior to cesarean section delivery to reduce the risk of uterine and wound infections in the mother (16), might offer an alternative explanation to the observed breast milk microbiota differences. Intrapartum antibiotic treatment is also recommended in selected cases in vaginal deliveries to reduce the risk of neonatal infections (17). Given the profound impact of antibiotic exposure on the intestinal microbiota (7), it is conceivable that maternal intrapartum antibiotic treatment may also perturb the breast milk microbiota. This notion is consistent with recent experimental studies suggesting that external factors including diet may influence the indigenous bacteria inhabiting the mammary epithelium (18). This is to our knowledge the first study in which the contribution of both delivery mode and antibiotic exposure to altered breast milk microbiota have been investigated.

Our results suggest that the mode of delivery has an independent impact on the microbial composition of breast milk. Cesarean section delivery was associated with reduced breast milk microbiota diversity and richness. Furthermore, 16 bacterial families detected in the breast milk of mothers who had delivered by vaginal delivery were not found in the breast milk of mothers who had delivered by cesarean section. The clinical significance of these findings obtained from a relatively small number of subjects is open to debate since the role of the bacteria in breast milk in mediating the beneficial effects of breastfeeding or determining the child's health remains poorly understood. Nonetheless, we and others have reported that the mode of birth markedly influences the developing gut microbiota both in the neonatal period and later in childhood (19–21). This may at least partially explain the epidemiological association between birth by cesarean section and increased incidence of non-communicable diseases later in life (22).

According to a recent report, breast milk microbes are an important source of colonizing microbes for the breastfed infant (5). It has been estimated that more than a quarter of the intestinal microbes in 1 month old breastfed infants originate from breast milk (5). It is therefore intriguing to hypothesize that the altered gut microbiota composition observed in infants born by cesarean section may in part be caused by differences in breast milk microbiota. Exposure to intrapartum antibiotic at delivery had an independent but relatively modest effect on breast milk microbiota at 1 month *postpartum*. Interestingly, antibiotic exposure appeared to increase bacterial richness and diversity in breast milk. The mechanism for this effect remains unknown. The microbial composition of the breast milk changes within the lactation state and intrapartum antibiotics may have had more significant effects on breast milk microbiota in earlier stages of lactation. Relying on milk samples obtained at only one time point is an obvious limitation of our study and future studies should assess the breast milk microbiota serially to obtain more reliable results. Confounding by dietary or other environmental exposures or maternal factors cannot be ruled out even though the study was designed to minimize such impact by including only healthy mothers with uncomplicated pregnancies (with the exception of allergic disease and gestational diabetes mellitus) and term or near term deliveries. It is also possible that some of the bacteria observed in this study *(e.g., Streptococcae, Staphylococcae)* may originate from maternal skin or even the environment. However, breast milk was detected to contain several anaerobic species such as *Bifibacteriaceae*, which are mostly found in the human intestine and not on the skin or environmental surfaces. We therefore interpret our results to provide an important addition to recent studies raising concerns about the safety of intrapartum antibiotic use.

The practice of antibiotic prophylaxis before cesarean section delivery is based on convincing evidence for decreased risk of uterine and wound infections in the mother (16). However, the authors of the Cochrane systematic review and meta-analysis demonstrating these benefits state that the impact of intrapartum prophylaxis on the child is unknown. According to a study by Azad et al. intrapartum antibiotic prophylaxis is associated with significant alterations in infant gut microbiota composition at the ages of 3 and 12 months as compared to non-exposed infants (23). We have reported based on a case-control study that intrapartum antibiotic exposure may be associated with increased occurrence of infantile colic (24). The extent to which these potentially detrimental effects are mediated by breast milk microbiota is currently not known. It is of note, however, that bacteria belonging to the genus *Bifidobacterium* were only found in the breast milk of mothers who had not received intrapartum antibiotics in this study. This observation may be of significance since reduced abundance of fecal bifidobacteria in early infancy has previously been associated with increased risk of non-communicable diseases including atopic disease and overweight in later life (25, 26).

The results of the present study demonstrate that both cesarean section delivery and intrapartum antibiotic treatment independently affect breast milk microbiota composition 1 month after delivery. These two exposures often cluster in the same mothers. The clinical significance of breast milk microbes was recently highlighted by our study indicating that infants receive bacterial antibiotic resistance genes via breastfeeding and exposure to intrapartum antibiotics may increase the abundance of specific antibiotic resistance genes in the infant gut (27). The contribution of altered breast milk microbiota to the gut microbiota perturbations and increased risk of non-communicable diseases associated with cesarean section delivery and early antibiotic exposure is a novel research question to be addressed in adequately powered clinical studies.

## Ethics Statement

This study was carried out in accordance with the recommendations of name of guidelines, name of committee with written informed consent from all subjects. All subjects gave written informed consent in accordance with the Declaration of Helsinki. The protocol was approved by the Ethics Committee of the Hospital District of Southwest Finland.

## Author Contributions

SR and EI designed the study and conducted the clinical trial. HH conducted the statistical analysis and wrote the first draft of manuscript. HK, SS, and MC conducted the microbial analysis of breast milk microbiota. All authors participated on writing the manuscript.

### Conflict of Interest Statement

The authors declare that the research was conducted in the absence of any commercial or financial relationships that could be construed as a potential conflict of interest.
