# Supplementary material for: Breast Milk Microbiota Is Shaped by Mode of Delivery and Intrapartum Antibiotic Exposure
Source: Front Nutr. 2019 Feb 4;6:4. doi: 10.3389/fnut.2019.00004 (PMC6369203; doi:10.3389/fnut.2019.00004)
Supplement: Supplementary file 1 [file Image-1.pdf]

Supplementary Figure 1A

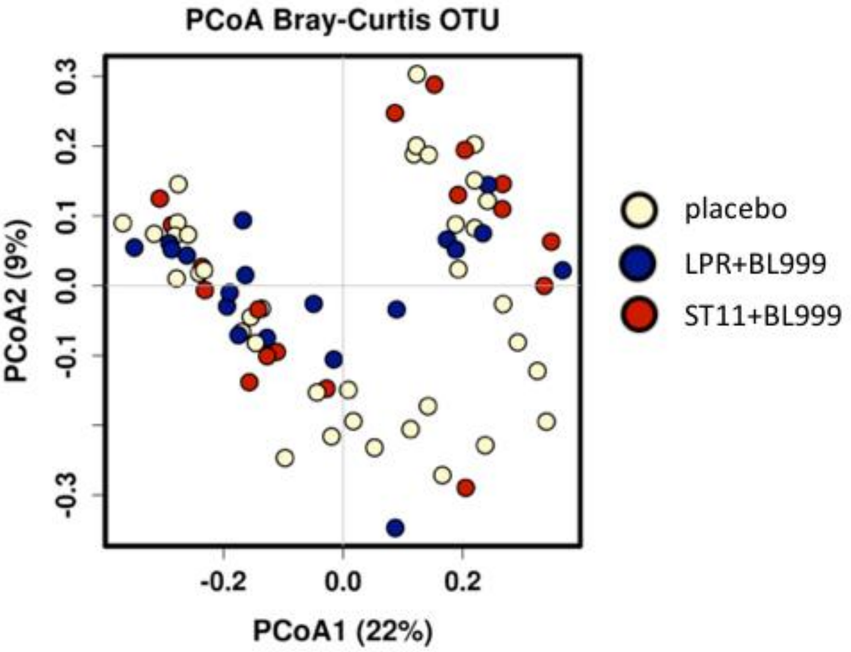

Supplementary Figure 1B

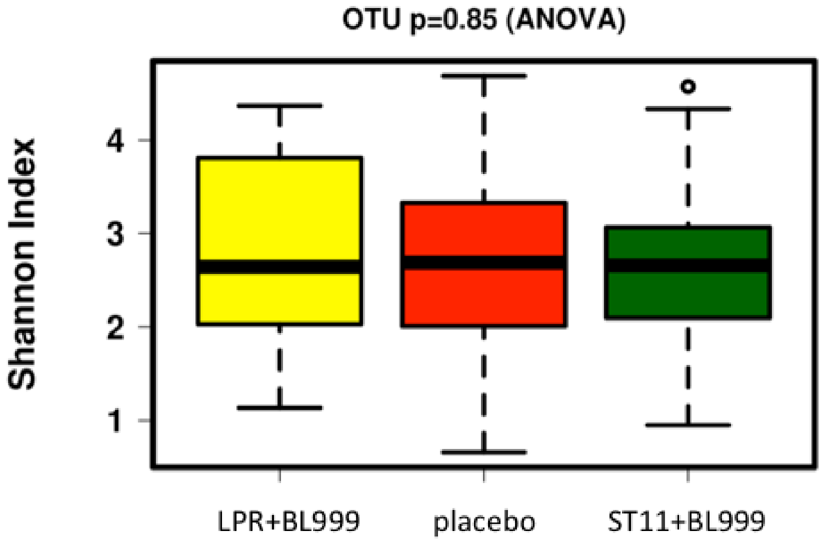

Supplementary Figure 1C

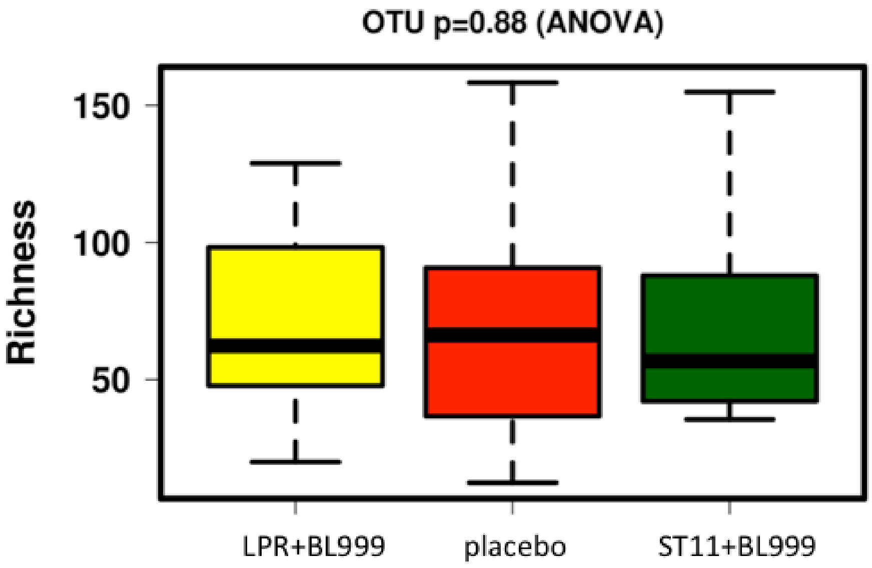

Supplementary Figure 1. Maternal probiotic supplementation with either the combination of *Lactobacillus rhamnosus* LPR and *Bifidobacterium longum* BL999 (LPR+BL999) or the combination of *Lactobacillus paracasei* ST11 and *Bifidobacterium longum* BL999 (ST11+BL999) was not associated with significant changes in the breast milk microbiota 1 month after delivery as compared to placebo. No clustering based on the intervention was detected by PCoA (ANOSIM p=0.96) (**A**), nor were there statistically significant differences in breast milk microbiota alpha diversity as assessed by the Shannon index (**B**) or in microbiota richness (**C**).
